# Book Review: The Psychological Experience of Integrating Content and Language

**DOI:** 10.3389/fpsyg.2021.671529

**Published:** 2021-04-27

**Authors:** Yingna Wang, Mateusz Marecki

**Affiliations:** College of Foreign Languages, Jilin University, Changchun, China

**Keywords:** psychological experience, integrating language and content, emotion, identity, well-being

## Summary of the Book

Integrated content and language (ICL) refers to programs where school subjects are taught in a language, usually English, that is not the learner's or teacher's first language. It encompasses several related approaches, including foreign language medium of instruction, bilingual education, and content and language integrated learning. As an innovative pedagogical trend that has been widely implemented internationally at all stages of education since the 2000s, these programs in the first place aim at developing students' language proficiency alongside expanding their content knowledge. The organization and implementation of ICL projects has been extensively documented in the existing literature. However, much of this research fails to acknowledge that the success of these programs depends in large part on less controllable psychological factors, such as the commitment, motivation, engagement, and attitudes of the participants involved. *The Psychological Experience of Integrating Content and Language* seeks to fill that gap.

This edited collection of papers brings together empirical studies with one main goal in mind: to identify the psychological processes and experiences of key stakeholders in diverse ICL settings. While discussing the challenges and opportunities that ICL programs may present to learners and teachers, it focuses on a range of important issues affecting classroom experiences, such as participants' changing identities, emotions, reactions, self-concepts, commitment, well-being, beliefs, attitudes, and self-efficacy.

The book consists of 16 chapters, which can be further divided into four thematic sections. Following the editors' introduction, Chapters 2–4 explore the importance of teachers' and students' identity and self-concept in ICL contexts. Chapters 5 through 8 look into teacher cognition and the beliefs of teachers and learners. Chapters 9–11 cluster around the concepts of well-being and confidence to discuss the challenges and opportunities key stakeholders face in ICL settings. The final section (Chapters 12–15) includes chapters on professional development, classroom implementation, interventions, and teacher research. The volume concludes with a chapter written by the editors to synthesize the key lessons and insights from the collection and suggests a future research agenda.

The contributors to the volume point out that ICL settings may be both the sites of growth and tensions for teachers and learners. They require a heavier workload and increased time investment on the part of the teachers. Some educators may experience linguistic insecurity or face threats to their professional integrity as they are expected to negotiate and juggle their multiple identities as content and language teachers. However, the conflicts and tensions that often emerge in ICL contexts may also serve as sites of development for teachers' relational identities. It is argued in the book that targeted training programs and supportive teacher networks may help bolster teachers' confidence and well-being.

As for students, they tend to feel anxiety and frustration in ICL settings when they are new to the program, lack confidence in their own language competence or are unable to meet their teachers' expectations. On the other hand, some have expressed positive emotions, having experienced less language learning anxiety than students in non-ICL contexts.

## Evaluation of the Book's Content

Overall, this book represents an impressive undertaking in that it teems with fresh ideas and is remarkable in its depth, breadth, and insight. First, it provides a wealth of perspectives to give a fuller picture of the way key stakeholders respond to ICL programs. Noteworthy, contrary to the tendency of overfocusing on learners' needs and preferences in the field of language learning psychology, 10 of the chapters foreground the teacher's perspective. Since students and teachers are significantly impacted by adopting ICL programs, it is crucial to appreciate and evaluate the effectiveness of learning and teaching methods in ICL programs from their perspectives.

Secondly, the collection reflects on the psychological experience of ICL in diverse educational and cultural settings. It features accounts of learners and educators from primary, secondary, and tertiary school settings, who come from such distant countries as Argentina, Austria, Canada, Finland, Spain, Japan, the United States, and Wales. As such, it offers a comprehensive overview of how ICL programs work globally.

Thirdly, this volume features studies that adopt a wide range of methodological approaches, most notably mixed methods research, including multiple interviews, classroom observations, video-taped lessons, or stimulated recall sessions. Thematic and narrative analysis were combined for qualitative analysis of interview data. Innovative methods were used, such as quantitative-qualitative methods, teacher research, a novel reflection that asked teachers to write a note to their former selves about the psychological and pedagogical aspects they wish they understood before starting their ICL teaching. Finally, a validated corpus-linguistics sentiment analysis tool SEANCE 1.2.0 (Crossley et al., 2017) was used to code feelings, emotions, social cognition, and social order.

## Discussion

As an insightful and thought-provoking contribution to the field of language teaching, this collection provides valuable guidance to researchers, teachers, and teacher educators who are interested in ICL programs, classroom teaching or/and psychology of language learning and teaching. Over the past two decades, the field of applied linguistics has witnessed a “social turn” and, more recently, an “emotional turn,” and has shifted its focus from pure cognitive approaches (White, 2018; Dewaele et al., 2019; Lantolf and Swain, 2019). In line with this trend, the book under review represents the diversity, complexities and dynamism of psychological experiences in teaching and learning in various ICL contexts.

It provides implications for research, practice, and professional development. Professional development programs should cultivate teachers' confidence in using their entire linguistic repertoire, target language, and language teaching pedagogy. In addition, a more contextualized approach is needed in designing meaningful professional development programs which address stakeholders' needs and challenges in situated settings. Further research should explore the questions of how, why and under what circumstances teachers embrace their identities, as well as the impact of their psychological experience on teaching and learning, as it is important to better explain what teachers do in classrooms and how they influence the ecology of classrooms.

## Author Contributions

YW: draft the review and revisions. MM: revisions. Both authors: contributed to the article and approved the submitted version.

## Conflict of Interest

The authors declare that the research was conducted in the absence of any commercial or financial relationships that could be construed as a potential conflict of interest.
